# *Ab initio* prediction for product stereo-specificity in the CH_3_CHI + O_2_ reaction: formation of *syn-* vs *anti-*CH_3_CHOO

**DOI:** 10.1007/s00894-025-06426-4

**Published:** 2025-07-11

**Authors:** Hue-Phuong Trac, Putikam Raghunath, Ming-Chang Lin

**Affiliations:** Department of Applied Chemistry and Center for Emergent Functional Matter Science, National Yang Ming Chiao Tung University, Hsinchu, 300 Taiwan

**Keywords:** Ab initio MO calculation, CH_3_CHI + O_2_, *syn*-CH_3_CHOO, *anti*-CH_3_CHOO

## Abstract

**Context:**

The stereo-specific production of *syn*- and *anti*-CH_3_CHOO conformers from the CH_3_CHI + O_2_ reaction has been investigated by ab initio quantum-chemical and statistical theory studies. The results of the studies clearly indicate that the [*syn*]:[*anti*] product ratio depends on both temperature and pressure of the reaction system, and is kinetically, rather than thermodynamically, controlled. Most experimental data measured near room temperature at 2–10 Torr He pressure agree with the predicted results in terms of either the absolute rate constants for *syn*- and *anti*-CH_3_CHOO production and/or the [*syn*]:[*anti*] product ratio. If the stereo-specificity of *syn*- and *anti*-CH_3_CHOO formation were controlled thermodynamically, one would predict [*syn*]:[*anti*] = 241:1 independent of pressure at 298 K, instead of (80 ± 10):(20 ± 10) measured experimentally or 86:14 predicted theoretically at 5-Torr He pressure.

**Methods:**

All calculations were performed using Gaussian 16 software. Geometry, frequency, and IRC analysis calculations were conducted at the B3LYP/Aug-cc-PVTZ level of theory. The potential energy surface of the system was computed at the CCSD(T)/Aug-cc-PVTZ//B3LYP/Aug-cc-PVTZ level. The rate constants for individual product channels in the reaction, including the direct production of IO + CH_3_CHO and the collisional deactivation of the excited CH_3_CHIO_2_* intermediate formed by the association of CH_3_CHI with O_2_, were predicted by statistical theory calculations using the Variflex code.

**Supplementary Information:**

The online version contains supplementary material available at 10.1007/s00894-025-06426-4.

## Introduction

Since the seminal work of Taatjies and coworkers [1] on the effective method for generation of the CH_2_OO from the CH_2_I + O_2_ reaction, there have been voluminous publications on the production, detection, and reactions of various Criegee intermediates (CIs) [2–7]. For the CIs with more than two carbon atoms, different conformers could be produced in the R_1_R_2_CI + O_2_ reactions. Take the CH_3_CHI + O_2_ reaction, for example; both *syn*-CH_3_CHOO and *anti*-CH_3_CHOO were detected in the reaction [2, 3, 7–15]. The relative CI product yields and their total rate constants have been reported in the referenced studies, with the [*syn*-CH_3_CHOO]:[*anti*-CH_3_CHOO] ratio ranging from 90:10 to 70:30, and the total rate constant in the range of 3.8 × 10^−12^ to 8.6 × 10^−12^ cm^3^ molecule^−1^ s^−1^ at the He pressure of 2–10 Torr measured near room temperature. The absolute rate constant for the CI product formation appears to be close to the known second-order kinetics for the combination of alkyl radicals with the molecular oxygen, typically in the range of 5 × 10^−12^–10^−11^ cm^3^ molecule^−1^ s^−1^ [16].

In this study, we are interested in the mechanism that controls the kinetics for the product stereo-specificity in the simpler CI reaction, CH_3_CHI + O_2_, which can generate different conformer products. If the newly formed C-O_2_ bond is as strong as that of the CH_3_-O_2_ bond, about 30 kcal mol^−1^ [17], one would expect a likely pressure-dependence in the observed [*syn*-CH_3_CHOO]:[*anti*-CH_3_CHOO] ratio and, particularly, the rate constants for their formation on account of the collisional quenching of the excited intermediate CH_3_CHIO_2_*. In addition, the production of CH_3_CHO + IO will competitively drain the yields of the two conformers. IO has been directly detected by Enami et al. [18] in the CH_3_CHI + O_2_ reaction with the absolute rate constant of 4 × 10^−13^ cm^3^ molecule^−1^ s^−1^ employing the sensitive cavity-ring-down kinetic spectrometric technique [19]. They ruled out the potential contribution of IO from the secondary reaction such as CH_3_CHO_2_ + I. Importantly, they also established at the density-functional theory level the transition states for various reactions of iodo-alkyl radicals with O_2_. For the CH_2_I and CH_3_CHI reactions with O_2_, the barriers for IO production were estimated to lie 3.3 and 8.6 kcal mol^−1^ below the reactants, suggesting the propensity for the direct formation of IO, which may affect competitively the yields of CH_3_CHOO conformers in the case of the CH_3_CHI + O_2_ reaction. Parenthetically, we should mention that Ting et al. [2] detected IO formation by transient UV absorption spectroscopy in the CH_2_I + O_2_ reaction. Through the simultaneous time-resolved detection of CH_2_I_2_, CH_2_OO, and IO, aided by kinetic modeling, they concluded that in the CH_2_I + O_2_ reaction system, IO was formed primarily by secondary reactions involving I, CH_2_OO, and CH_3_CHIO_2_ [2]. Furthermore, if the C-O_2_ torsional vibrations in the CH_3_CHIO_2_ intermediate are highly hindered, one would also expect the production of two different conformers of the CH_3_CHIO_2_ intermediate, which can respectively give rise to the *syn*- and *anti*-CH_3_CHOO conformers. The different competitive processes mentioned above can be reasonably elucidated and accounted for through the quantum-chemical study of the potential energy surface involved, aided by reliable statistical-theory calculations as reported herein.

### Computational details

#### Ab initio calculations

The mechanism for the reaction of CH_3_CHI with O_2_ has been studied by high-level quantum-chemical calculations. Electronic structure calculations were carried out with the Gaussian 16 program package [20]. The B3LYP density functional theory [21] with the Aug-cc-PVTZ basis set [22, 23] was employed to optimize the geometries of the reactants, intermediates, and products. The Aug-cc-PVTZ-pp basis set [24] was used to calculate the Iodine atom, incorporating a relativistic pseudopotential that largely accounts for scalar relativistic effects in Iodine. Vibrational frequencies of all species involved were calculated at the same level of theory for optimization as well as the normal mode analyses. All stationary points were identified by the number of imaginary frequencies (NIMG) with NIMG = 0 for stable species and NIMG = 1 for transition states. To obtain more reliable energetics, single-point energies based on the B3LYP/Aug-cc-PVTZ geometries were calculated at the CCSD(T)/Aug-cc-PVTZ level [25].

#### Rate constant predictions

The kinetics for the reaction of CH_3_CHI with O_2_, which contains different types of elemental steps as mentioned in the “Introduction” section, was predicted with the versatile Variflex program [26] based on the transition state theory (TST) and Rice-Ramsperger-Kassel-Marcus (RRKM) theory [27]. For reaction steps with well-defined transition states, TST was employed to predict their rate constants. For the reaction steps without well-defined TSs, i.e., the barrierless reaction channels, such as CH_3_CHI + O_2_ → CH_3_CHIO_2_, the variational TST was used to compute their association and dissociation rate constants [26]. For these processes, their potential functions or minimum energy paths (MEPs) were estimated to cover a wide range of separations. The effect of pressure on the association and decomposition processes, the RRKM theory embedded in the Variflex code, was utilized.

To more reliably predicted the effect of pressure due to collisional quenching of the excited CH_3_CHIO_2_ by the diluent, He, as employed in all experiments, the Lennard–Jones (LJ) potential for the CH_3_CHIO_2_-He interaction was computed at the B3LYP(D3)/Aug-cc-PVTZ level of theory. The fitting of the computed data to the equation, *V*(*r*) = 4ε [(σ/*r*)^12^ − (σ/*r*)⁶], giving ε = 15.43 K and σ = 3.60 Å as shown in Fig. S3. These parameters will be used to calculate the L-J collision frequency *Z*_LJ_. The efficiency for He quenching was estimated with the exponential down model with the average downward energy transferred per collision < ΔE > _down_ = 70 cm^−1^.

## Results and discussions

### Potential energy surfaces and the mechanism of the CH_3_CHI + O_2_ reaction

**Fig. 1:**
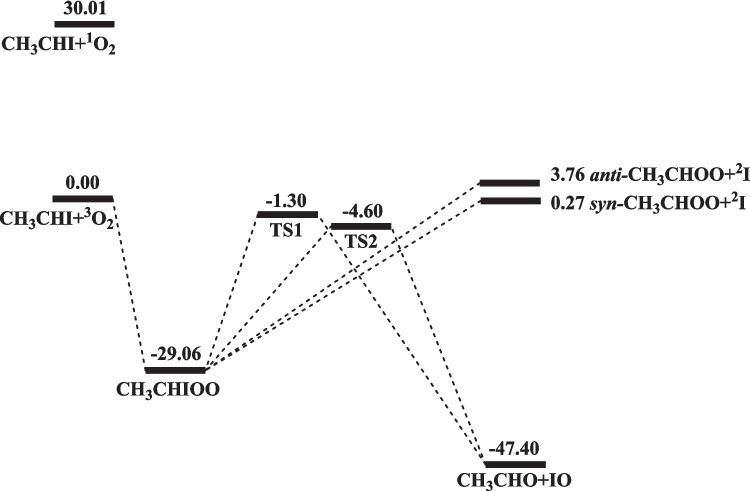
The potential energy surface of the CH_3_CHI + O_2_ reaction computed at the CCSD(T)/Aug-cc-PVTZ//B3LYP/Aug-cc-PVTZ level (energy in kcal mol^−1^)

For the elucidation of the kinetics and mechanism of the CH_3_CHI reaction with O_2_ in the gas phase, we have established the potential energy surfaces (PES) of the reaction at the CCSD(T)/Aug-cc-PVTZ//B3LYP/Aug-cc-PVTZ level of theory as aforementioned. If the C-O_2_ torsional vibration in the CH_3_CHIO_2_ association intermediate is highly hindered, one would expect the formation of two distinct conformers (LM1 and LM2) as shown in Fig. S1 in the Supporting Information section. These conformers can respectively give rise to the *syn-* and *anti*-CH_3_CHOO products by breaking their C-I bonds without well-defined transition states as shown in the figure. The elimination of IO from these conformers via 4-centered TSs, however, takes place with well-defined TS1 and TS2 from LM1 and LM2, respectively, with their structures given in Fig. S2. If the predicted C-O_2_ torsional vibration frequencies are low (79 and 74 cm^−1^, respectively) as presented in Table S1, the PES depicted in Fig. S1 for the reaction can be simplified as shown in Fig. 1 with one reaction intermediate containing the free C-O_2_ torsional vibration. In a complete internal rotation, the intermediate goes via the LM1 and LM2 structure once. The geometries optimized at the B3LYP/Aug-cc-PVTZ level for various species are presented in Fig. S2, while the energies of the species appeared in Fig. 1 are summarized in Table 1. To evaluate the suitability and reliability of the single-reference wave function employed in the CCSD(T) calculations, we have carried out T1 diagnostic analyses for all the species involved in the reaction including the reactants, intermediates, transition states, and products. The results show that all the opened shell species (the reactants, intermediates, transition states, I and IO) are within 0.045 as shown in Table S3. However, for the single products, *syn*- and *anti*- CH_3_CHOO both have the diagnostic values of 0.036, which may be compared with the value for CH_2_OO (0.042), computed at the CCSD(T)/aug-cc-pVTZ//M06-2X/aug-cc-pV(T + d)Z level [28]. Both values are greater than 0.02 as recommended for the singlet species [29]. The higher T1 values for the CIs may be attributed to their zwitterionic character of the species. The energies predicted at the CCSD(T)/Aug-cc-PVTZ//B3LYP/Aug-cc-PVTZ level of theory, as will be shown in the following, are consistent with the thermochemistry recommended recently for the CH_3_CHOO conformers as well as the predicted kinetics for the formation of the conformers.

**Table 1:** The heat of CH_3_CHI + O_2_ reaction at 0 K of calculated at the B3LYP/Aug-cc-PVTZ and CCSD(T)/Aug-cc-PVTZ//B3LYP/Aug-cc-PVTZ levels

**Reaction**	B3LYP/Aug-cc-PVTZ	CCSD(T)/Aug-cc-PVTZ//B3LYP/Aug-cc-PVTZ
CH_3_CHI + ^3^O_2_ = *syn-*CH_3_CHOO + ^2^I	− 1.37	0.27
CH_3_CHI + ^3^O_2_ = *anti-*CH_3_CHOO + ^2^I	1.28	3.76
CH_3_CHI + ^3^O_2_ = CH_3_CHO + IO	− 48.86	− 47.40
CH_3_CHIOO	− 23.42	− 29.06
TS1	− 2.13	− 1.30
TS2	− 6.33	− 4.60

Values are given in units of kcal mol^−1^

As depicted in Fig. 1, the reaction occurs to form the CH_3_CHIO_2_ intermediate with a binding energy of 29.1 kcal mol^−1^. CH_3_CHIO_2_ can fragment to give *syn-*CH_3_CHOO + I and *anti-*CH_3_CHOO + I with energies 0.3 and 3.8 kcal mol ^−1^ above the reactants, respectively. The result suggests that the formation of *syn*-CH₃CHOO is energetically more favorable over the *anti-*CH_3_CHOO conformer. The kinetics of their formation is, however, controlled by the exit variational TSs as will be discussed in the following. The two 4-member-ring transition states, TS1 and TS2, shown in Fig. S2 for the formation of CH_3_CHO + IO locate below the reactants at 1.3 and 4.6 kcal mol^−1^, respectively. The reactions are exothermic by 47.4 kcal mol^−1^. These energetics together with the vibrational frequencies and molecular parameters summarized in Table S1 will be employed for rate constant predictions using the Variflex code as aforementioned.

### Rate constant predictions

The CH_3_CHI + O_2_ reaction can be mechanistically described as follows:

$$\begin{aligned}CH_3CHI+O_2\leftrightharpoons CH_3CHIO_{2}^{*}&\rightarrow syn-CH_3CHOO+I\\&\rightarrow anti-CH_3CHOO+I\\&\rightarrow CH_3CHO+IO\left(via\;TS1\right)\\&\rightarrow CH_3CHO+IO\left(via\;TS2\right)\\&\rightarrow CH_3CHIO_2\left(+M\right)\end{aligned}$$

In the above scheme, “*” represents internal excitation of the CH_3_CHIO_2_ intermediate through the formation of the C-O_2_ bond with energy ≥ 29.1 kcal mol^−1^. In the last reaction step, (+ M) represents collisional quenching with the third body M, He. The second-order rate constants for the above reaction steps may be given by *k*_1_, *k*_2_, *k*_IO1_, *k*_IO2_, and *k*_M_, respectively.

The kinetics for the full reaction scheme can be reliably computed with the Variflex code [26] written on the basis of statistical TST and RRKM theories as aforementioned. For the initial association producing CH_3_CHIO_2_* and the fragmentation of CH_3_CHIO_2_* producing *syn*- and *anti*-CH_3_CHOO taking place barrierlessly, the variational TST (VTST) based on the predicted MEPs was employed for rate constant calculations. Take the CH_3_CHI + O_2_ association reaction, for example; its MEP was established by varying the CH_3_CHI and O_2_ separation from 1.43 to 5.93 Å with a step size of 0.1 Å at the B3LYP/Aug-cc-PVTZ level. The Morse function, ${{V}}\left( {{r}} \right){{ = D}}_{{{e}}} \left[ {\left( {{{1 - e}}^{{{{ - \beta }}\left( {{{r - r}}_{{{e}}} } \right)}} } \right)^{{2}} { - 1}} \right]$, was utilized to represent the MEP obtained by full optimization along the varying reaction coordinate. Here, *D*_e_, *R*, and *R*_e_ have the usual meanings. The predicted Morse function for the CH_3_CHIOO → CH_3_CHI + O_2_ MEP can be represented by *β* = 3.5 Å^−1^ with the values of *D*_e_ presented in Fig. 1 (with the zero-point energy included in the MEP). Similarly, for the production of the *syn*- and *anti*-CH_3_CHOO conformers by breaking the C-I bond, their variational MEPs could be represented by the Morse functions with the *β* values, 2.5 Å^−1^ and 2.6 Å^−1^, with the corresponding *D*_e_ values, respectively. For the production of IO from CH_3_CHIO_2_*, the vibrational frequencies and moments of inertia of the two distinct TSs are presented in Table S1 and their energies given in Fig. 1 and Fig. S1.

**Fig. 2:**
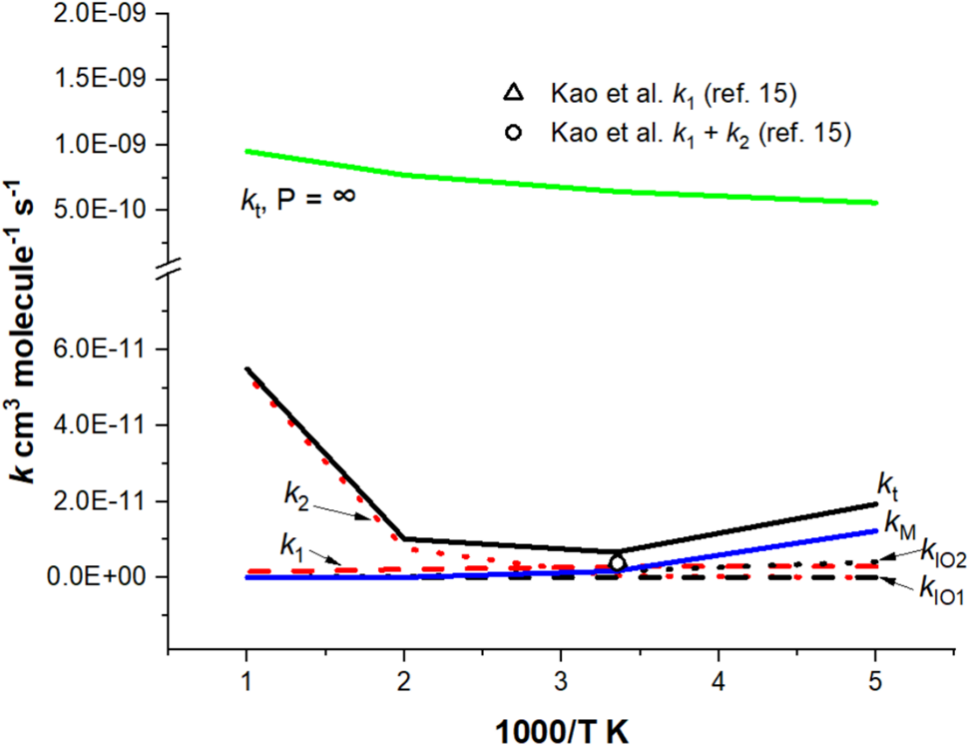
Temperature effect on the predicted rate constants and comparison with experimental data in the He bath gas at 298 K for the CH_3_CHI + O_2_ reaction forming *syn-*CH_3_CHOO (*k*_1_) and *anti-*CH_3_CHOO (*k*_2_)

Figure 2 presents the theoretically predicted temperature dependence of the individual step rate constants (*k*_1_, *k*_2_, *k*_IO1_, *k*_IO2_, and *k*_M_) at 5 Torr He pressure and the total rate constants at 5 Torr as well as at the infinite pressure limit, at which the reaction produces *only* the fully deactivated or thermalized reaction intermediate, CH_3_CHIO_2_ with total rate constant *k*_t_ = *k*_M_ = Z_LJ_ [M]. The experimental data obtained for 5 Torr He at 298 K for *k*_1_ and *k*_1_ + *k*_2_ reported by Kao et al. [15] are also shown in the figure for comparison. Table 2 numerically presents the individual step rate constants predicted for several specific pressures studied experimentally near room temperature for comparison with the available experimental data obtained for *k*_1_, *k*_2_, *k*_1_ + *k*_2_, and/or *k*_1_/*k*_2_. *The theoretical results clearly indicate that k*_1_ > *k*_2_*, k*_IO2_ > *k*_IO1,_*and the collisional quenching rate constant k*_M_* producing CH*_3_*CHIO*_2_* is having the same order of magnitude as k*_1_ and *k*_IO2_. *Furthermore, the theoretical values for k*_1_*, k*_2_*, k*_1_ + *k*_2_*, and/or k*_1_/*k*_2_*agree with most experimental data*, particularly with the most recent finding of Kao et al. [15] studied at 298 K with 5 Torr He buffer gas: *k*_1_ = 2.55 ± 0.5, *k*_2_ = 0.64 ± 0.1, *k*_1_ + *k*_2_ = 3.8 ± 0.7 (in units of 10^−12^ cm^3^ molecule^−1^ s^−1^), and *k*_1_/*k*_2_ = (80 ± 10):(20 ± 10). These values compare very closely with the theoretical result, *k*_1_ = 2.87, *k*_2_ = 0.45, *k*_1_ + *k*_2_ = 3.33 (in units of 10^−12^ cm^3^ molecule^−1^ s^−1^), and *k*_1_/*k*_2_ = 86:14, respectively. We should, however, point out that our predicted results differ noticeably from experiments in two cases as indicated in Table 2. At 295 K, 2 Torr He pressure, Howes et al. [3] reported *k*_1_ + *k*_2_ = (8.6 ± 2.2) × 10^−12^ cm^3^ molecule^−1^ s^−1^, which is four times bigger than what we predicted, 2.15 × 10^−12^ cm^3^ molecule^−1^ s^−1^, although their *k*_1_/*k*_2_ = (86 ± 11:14 ± 11) ratio agrees well with the predicted result *k*_1_/*k*_2_ = 80:20. In the 2nd case, at 293 K and 10 Torr He, Sheps et al. [10] obtained *k*_1_/*k*_2_ = 70:30 with *k*_1_ + *k*_2_ = (8.0 ± 0.8) × 10^−12^ cm^3^ molecule^−1^ s^−1^; the *k*_1_/*k*_2_ ratio differs greatly from the predicted value 92:8, although the theoretical sum of rate constants *k*_1_ + *k*_2_ = 5.3 × 10^−12^ cm^3^ molecule^−1^ s^−1^ agrees reasonably with the reported value. We should also point out that the collisional quenching rate constant *k*_M_ decreases rapidly with temperature as indicated in Fig. 2 and Table S2.

**Table 2:** Comparison of predicted and experimental rate constants (in units of 10^−12^ cm^3^ molecule^−1^ s^−1^) for the CH_3_CHI + O_2_ reaction in He under experimental conditions

***T* (K)**	***P* (Torr)**	***k***_**M**_	***k***_**1**_	***k***_**2**_	***k***_**IO1**_	***k***_**IO2**_	***k***_**1**_ + ***k***_**2**_	***k***_**1**_:***k***_**2**_
295	2	0.76	1.73	0.42	0.02	2.10	2.15	80:20
		-	-	-	-	-	(8.60 ± 2.2)^a^	(86 ± 11:14)^a^
298	4	1.43	2.50	0.45	0.02	1.75	2.95	85:15
		-	-	-	-	-	-	(90:10)^b^
298	5	1.74	2.87	0.45	0.02	1.66	3.33	86:14
		-	(2.55 ± 0.5)^c^	(0.64 ± 0.1)^c^	-	-	(3.80 ± 0.7)^c^	(80 ± 10:20 ± 10)^c^
293	10	3.46	4.91	0.41	0.02	1.48	5.31	92:8
		-	-	-	-	-	(8.00 ± 0.8)^d^	(70:30)^d^

Experimental values are given in parentheses

^a^ref. 3; ^b^ref. 9; ^c^ref. 15; ^d^ref. 10

**Fig. 3:**
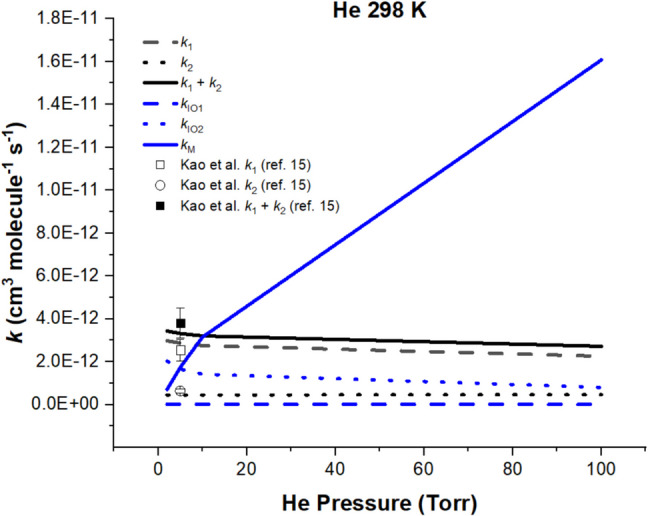
Pressure effect on the predicted rate constants and comparison with experimental data in the He bath gas at 298 K for the CH_3_CHI + O_2_ reaction forming *syn-*CH_3_CHOO (*k*_1_) and *anti-*CH_3_CHOO (*k*_2_)

Figure 3 graphically shows the effect of the He pressure on individual rate constants including that for the deactivation of the excited intermediate CH_3_CHIO_2_* (*k*_M_). The pressure was varied from 2 to 100 Torr at 298 K, around which most experiments were carried out. It is interesting to note that all individual product channels varied weakly with P, except the second-order quenching rate constants *k*_M_, which increases linearly with P or [M], the concentration of the quencher He, as one would expect. The effect of pressure predicted at 298 K is also numerically presented in Table S2 for a clearer examination. From the table, *k*_1_ is seen to be slightly decreased with P while *k*_2_ remains essentially the same for 2 to 100 Torr, reflecting the 3.5 kcal mol^−1^ higher exit barrier for *k*_2_. The *k*_1_:*k*_2_ ratio is noted to vary weakly from 86.9:13.1 at 2 Torr to 82.9:17.1 at 100 Torr. Table S2 also summarizes the results for the effect of temperature on the individual rate constants for He pressure at 5 and 10 Torr with the temperature changing from 200 to 1000 K. *k*_1_ is noted to decrease steadily with T at both pressures, whereas *k*_2_ increases more drastically with T, again reflecting the 0.3 kcal mol^−1^ vs. 3.8 kcal mol^−1^ endothermicity, respectively. The opposite effects make the sum of rate constants, *k*_1_ + *k*_2_ decreasing less drastically with the increasing T. The opposite effects, however, magnify the effect of T on the *k*_1_:*k*_2_ ratio; at *P* = 5 Torr, *k*_1_:*k*_2_ = 99.5:0.5 at 200 K changes drastically to *k*_1_:*k*_2_ = 0.8:99.2 at 1000 K. The trend for the 10 Torr case is similar, changing from 99.4:0.6 at 200 K to 2.7:97.3 at 1000 K. *The intricate effects of pressure and temperature on k*_1_*, k*_2_*, k*_1_ + *k*_2_*, and k*_1_*/k*_2_* clearly reflect the fact that the stereo-specific CH*_*3*_*CHOO product formation in the CH*_3_*CHI* + *O*_2_* reaction is kinetically, rather than thermodynamically, controlled. In addition, the good agreement between the predicted values of k*_1_*, k*_2_*, k*_1_ + *k*_2_*, and the product ratio k*_1_*/k*_2_*also suggests the energies of syn- and anti-CH*_*3*_*CHOO predicted at the CCSD(T)/Aug-cc-PVTZ//B3LYP/Aug-cc-PVTZ level of theory are reliable*. In fact as alluded to above, the difference in the energies of *syn*- and *anti*-CH_3_CHOO conformers, as shown in Fig. 1 and Table 1, 3.49 kcal mol^−1^, agrees quantitatively with the recently recommended value, 3.46 ± 0.20 kcal mol^−1^ [30].

If the production of *syn*- and *anti*-CH_3_CHOO products were thermodynamically controlled by the Gibbs free energies of formation of the two conformers, *k*_1_*, k*_2_*, k*_1_ + *k*_2_*, and k*_1_*/k*_2_ should be pressure-independent and the [*syn*]:[*anti*] ratio predicted at 298 K with the Gibbs free energies of formation of the 2 conformers would be 241:1, which is very different from the kinetically predicted result 86:14 as well as the measured value (80 ± 10):(20 ± 10) at 5 Torr He pressure (Table 2).

Under the experimental conditions carried out near room temperature at 5 Torr He pressure [15], the reaction can be schematically described below with the predicted product branching ratios:
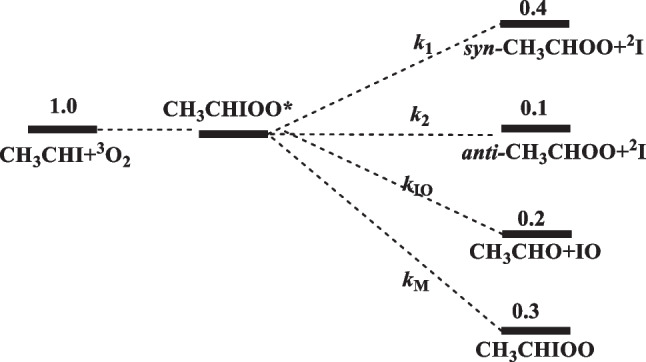


The result clearly indicates that the rates of formation of *syn*- and *anti*-CH_3_CHOO are comparable to those of IO production as well as the collisional stabilization of the excited CH_3_CHIOO* intermediate.

## Conclusions

The stereo-specificity of product formation in the CH_3_CHI + O_2_ reaction giving *syn-* and *anti*-CH_3_CHOO conformers has been investigated quantum-chemically with the statistical-theory prediction of rate constants for the two and other competing individual reaction channels. The system’s potential energy surface was characterized at the CCSD(T)/Aug-cc-PVTZ level of theory based on the geometries of all species involved computed with the B3LYP/Aug-cc-PVTZ method. The total rate constant for the reaction was found to be controlled by the initial association of the CH_3_CHI radical with O_2_, taking place variationally without a well-defined transition state. The excited association intermediate CH_3_CHIO_2_* can undergo fragmentation reactions producing the *syn-* and *anti*-CH_3_CHOO products by breaking the C-I bond; the process also occurs barrierlessly without well-defined transition states. The CH_3_CHIO_2_* intermediate can also decompose by two four-centered transition states giving CH_3_CHO + IO. In addition, the collisional quenching of CH_3_CHIO_2_* giving rise to thermalized CH_3_CHIO_2_ also happens competitively. These latter three processes compete effectively with the formation of the CH_3_CHOO conformers.

Under the conditions experimentally carried out at 2–10 Torr He pressure near room temperature, the predicted major rate constants for CH_3_CHOO and IO production and the collisional quenching of CH_3_CHIO_2_* are comparable. The theoretical [*syn*]:[*anti*] ratio at 298 K and 5 Torr He, 86:14, for example, agrees quantitatively with the latest, measured ratio, (80 ± 10):(20 ± 10); similarly, the magnitudes of the individual rate constants for the *syn*- and *anti*-CH_3_CHOO production agree closely with the experimental values. At 298 K, if the system pressure is increased from 2 to 10 Torr to 100 Torr, the individual rate constants for the two-conformer production (*k*_1_ and *k*_2_) and the [*syn*]:[*anti*] ratio (*k*_1_:*k*_2_) decline weakly with pressure (see Fig. 3 and Table S2), although the quenching rate constant *k*_M_ increased by a factor of 20. The result suggests that the population distribution of CH_3_CHIO_2_* near the dissociation limits are not strongly affected by the quenching of the excited intermediate at 298 K up to 100 Torr He pressure. At the high-pressure limit (*P* = ∞), however, all CH_3_CHIO_2_* can be effectively thermalized and no CH_3_CHOO and IO products can be formed.

The result of temperature effects on individual product channel rate constants including *k*_M_ indicates very drastic changes in the values of *k*_1_, *k*_2_, and *k*_M_. As illustrated by the result shown in Table S2, when the temperature of the system increases from 200 to 1000 K under 5 Torr or 10 Torr He pressure, the values of *k*_1_ decrease by a factor of 7 and 2 respectively, while the values of *k*_2_ increases by as much as 3.5 × 10^3^ times, reflecting the smaller exit barrier for the *syn*-conformer formation (0.3 kcal mol^−1^) and the higher barrier for the *anti*-conformer (3.8 kcal mol^−1^). Even more drastic change in the quenching rate constant is noted with *k*_M_ decreases by more than seven orders of magnitude. The combined effect causes the [*syn*]:[*anti*] ratio drastically changes from 99.5:0.5 at 200 K to 0.2:99.8 at 1000 K under 5 Torr He pressure, and 99.4:0.6 at 200 K to 2.7:97.3 at 1000 K under 10 Torr He pressure. The stereo-specificity of the *syn*- vs. *anti*-CH_3_CHOO formation is, therefore, clearly illustrated to be kinetically, rather than thermodynamically, controlled. If the stereo-specificity were thermodynamically controlled, [*syn*]:[*anti*] = 241:1 at 298 K, independent of pressure. The ratio differs greatly from the experimental result, (86 ± 11:14 ± 11), and the predicted value, 86:14 at 298 K and 5 Torr He pressure.

## Supplementary Information

Below is the link to the electronic supplementary material.

ESM 1 DOCX (1.68 MB)

## Data Availability

No datasets were generated or analysed during the current study.
