# Supplementary material for: Ab initio prediction for product stereo-specificity in the CH3CHI + O2 reaction: formation of syn- vs anti-CH3CHOO
Source: J Mol Model. 2025 Jul 11;31(8):205. doi: 10.1007/s00894-025-06426-4 (PMC12254064; doi:10.1007/s00894-025-06426-4)
Supplement: Supplementary file 1 — DOCX (1.68 MB) [file 894_2025_6426_MOESM1_ESM.docx]

**Supporting Information**

***Ab initio* prediction for product stereo-specificity in the CH_3_CHI + O_2_ reaction: Formation of *syn-* vs *anti-*CH_3_CHOO**

Hue-Phuong Trac, Putikam Raghunath and Ming-Chang Lin *

Department of Applied Chemistry and Center for Emergent Functional Matter Science

National Yang Ming Chiao Tung University, Hsinchu, Taiwan 300

***Corresponding authors:** [chemmcl@emory.edu](mailto:chemmcl@emory.edu)

**Fig. S1.** The potential energy surface of the CH_3_CHI + O_2_ reaction computed at the CCSD(T)/Aug-cc-PVTZ//B3LYP/Aug-cc-PVTZ level (energy in kcal/mol).

**Fig. S2.** The optimized geometries of the reactants, intermediates, transition states, and products of the CH_3_CHI + O_2_ reaction computed at the B3LYP/Aug-cc-PVTZ level


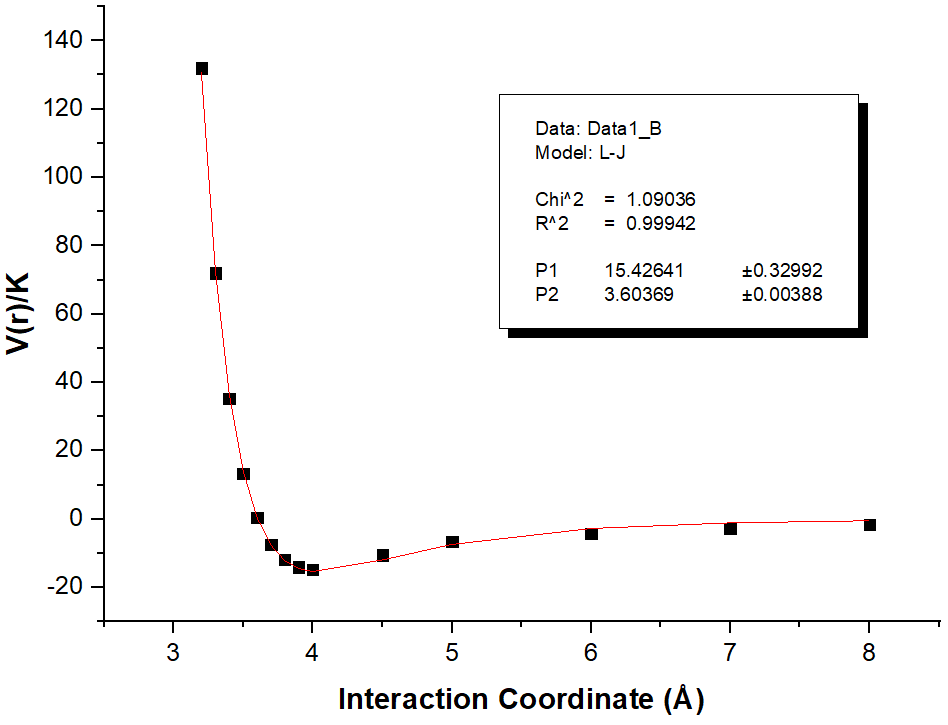


**Fig. S3.** The Lennard-Jones potential for the CH_3_CHIO_2_-He interaction computed at the B3LYP(D3)/Aug-cc-PVTZ level. Fitting of the cpomputed points to the L-J potential, V(r) = 4ε [(σ/r)¹² - (σ/r)⁶], gave ε = 15.43 K and σ = 3.60 Å.

**Table S1.** Vibrational frequencies and moments of inertia for reactants, intermediates, transition states, and products of the CH_3_CHI + O_2_ computed at the B3LYP/Aug-cc-PVTZ level

| Species or  Transition states | Moments of inertia I_A_, I_B_, I_C_ (a.u) | Vibrational Frequencies (cm^-1^) |
| --- | --- | --- |
| CH_3_CHI | 45.1, 604.5, 638.0 | 134, 264, 335, 552, 993, 998, 1098, 1243, 1403, 1460, 1477, 2967, 3040, 3080, 3206 |
| ^3^O_2_ | 0.0, 41.5, 41.5 | 1626 |
| *syn-*CH_3_CHIOO | 329.8, 1214.3, 1396.3 | 79, 227, 244, 261, 320, 501, 633, 800, 977, 1065, 1111, 1127, 1228, 1370, 1412, 1476, 1490, 3036, 3113, 3136, 3152 |
| *anti-*CH_3_CHIOO | 389.8, 1144.8, 1473.9 | 74, 221, 238, 254, 349, 488, 561, 833, 982, 1070, 1110, 1175, 1227, 1334, 1417, 1480, 1487, 3037, 3107, 3132, 3148 |
| TS1 | 350.1, 1131.8, 1309.1 | -243, 148, 193, 212, 303, 382, 604, 821, 872, 915, 1046, 1152, 1280, 1393, 1432, 1472, 1490, 2980, 3114, 3139, 3185 |
| TS2 | 381.3, 1117.4, 1446.5 | -146, 153, 169, 263, 289, 403, 530, 838, 881, 940, 1037, 1156, 1284, 1398, 1422, 1473, 1486, 3016, 3101, 3146, 3187 |
| TS3 | 399.9, 1025.3, 1350.8 | -117, 186, 243, 254, 399, 446, 632, 768, 974, 1055, 1091, 1151, 1249, 1327, 1411, 1481, 1486, 3038, 3103, 3113, 3134 |
| *syn-*CH_3_CHOO | 101.0, 256.2, 346.5 | 188, 296, 462, 675, 758, 903, 983, 1047, 1111, 1349, 1400, 1438, 1461, 1559, 3015, 3049, 3140, 3179 |
| *anti-*CH_3_CHOO | 36.2, 408.1, 433.2 | 160, 258, 324, 554, 868, 893, 968, 1067, 1157, 1344, 1415, 1459, 1463, 1577, 3020, 3062, 3132, 3146 |
| CH_3_CHO | 31.4, 177.5, 197.8 | 158, 510, 775, 886, 1129, 1136, 1379, 1422, 1460, 1469, 1805, 2870, 3022, 3073, 3135 |
| IO | 0.0, 183.1, 183.1 | 647 |

**Table S2.** The predicted rate constants and branching ratios at different temperatures and pressures in the He bath gas for the CH_3_CHI + O_2_ reaction forming *syn-*CH_3_CHOO (*k*_1_) and *anti-*CH_3_CHOO (*k*_2_)

| **T (K)** | **P (Torr)** | ***k*_M_** | ***k*_IO_** | ***k*_1_** | ***k*_2_** | ***k*_1_** + ***k*_2_** | ***k*_1_ : *k*_2_** |
| --- | --- | --- | --- | --- | --- | --- | --- |
| 298 | 2 | 7.13E-13 | 2.05E-12 | 2.98E-12 | 4.51E-13 | 3.43E-12 | 86.9 : 13.1 |
|  | 4 | 1.43E-12 | 1.77E-12 | 2.90E-12 | 4.51E-13 | 3.35E-12 | 86.6 : 13.4 |
|  | 5 | 1.74E-12 | 1.68E-12 | 2.87E-12 | 4.51E-13 | 3.32E-12 | 86.4 : 13.6 |
|  | 10 | 3.15E-12 | 1.44E-12 | 2.76E-12 | 4.52E-13 | 3.21E-12 | 85.9 : 14.1 |
|  | 100 | 1.61E-11 | 8.20E-13 | 2.26E-12 | 4.66E-13 | 2.72E-12 | 82.9 : 17.1 |
| 200 | 5 | 1.23E-11 | 4.20E-12 | 2.88E-12 | 1.50E-14 | 2.89E-12 | 99.5 : 0.5 |
| 300 |  | 1.68E-12 | 1.65E-12 | 2.79E-12 | 4.73E-13 | 3.27E-12 | 85.5 : 14.5 |
| 500 |  | 4.48E-14 | 2.89E-13 | 6.39E-13 | 7.64E-12 | 8.28E-12 | 7.7 : 92.3 |
| 1000 |  | 2.29E-19 | 4.85E-14 | 4.10E-13 | 5.33E-11 | 5.37E-11 | 0.8 : 99.2 |
| 200 | 10 | 1.96E-11 | 3.22E-12 | 2.62E-12 | 1.52E-14 | 2.63E-12 | 99.4 : 0.6 |
| 300 |  | 3.03E-12 | 1.41E-12 | 2.75E-12 | 4.74E-13 | 3.23E-12 | 85.3 : 14.7 |
| 500 |  | 9.17E-14 | 2.73E-13 | 2.12E-12 | 7.64E-12 | 9.76E-12 | 21.7 : 78.3 |
| 1000 |  | 5.65E-19 | 4.85E-14 | 1.50E-12 | 5.33E-11 | 5.48E-11 | 2.7 : 97.3 |

**Table S3.** T1 diagnostic values for reactants, intermediates, transition states, and products of the CH_3_CHI + O_2_ reaction computed at the CCSD(T)/Aug-cc-PVTZ//B3LYP/Aug-cc-PVTZ level

| Species or Transition states | T1 diagnostic values |
| --- | --- |
| CH_3_CHI | 0.014 |
| ^3^O_2_ | 0.018 |
| *syn-*CH_3_CHIOO | 0.022 |
| *anti-*CH_3_CHIOO | 0.022 |
| TS1 | 0.033 |
| TS2 | 0.032 |
| TS3 | 0.029 |
| *syn-*CH_3_CHOO | 0.036 |
| *anti-*CH_3_CHOO | 0.036 |
| CH_3_CHO | 0.014 |
| IO | 0.041 |
| I | 0.041 |
